# *Hairpin in a haystack*: *In silico* identification and characterization of plant-conserved microRNA in Rafflesiaceae

**DOI:** 10.1515/biol-2022-1033

**Published:** 2025-01-27

**Authors:** Adhityo Wicaksono, Karlia Meitha, Kiew-Lian Wan, Mohd Noor Mat Isa, Arli Aditya Parikesit, Jeanmaire Molina

**Affiliations:** Genomik Solidaritas Indonesia (GSI) Lab, Jl. Sultan Agung no. 29, Guntur, Jakarta, 12980, Indonesia; Biosciences and Biotechnology Research Center, Institut Teknologi Bandung, Jl. Ganesha no. 10, Bandung, 40132, Indonesia; Research group of Genetics and Molecular Biology, School of Life Sciences and Technology, Institut Teknologi Bandung, Jl. Ganesha no. 10, Bandung, 40132, Indonesia; Department of Biological Sciences and Biotechnology, Faculty of Science and Technology, Universiti Kebangsaan Malaysia, UKM Bangi, Selangor, 43600, Malaysia; Malaysia Genome and Vaccine Institute, National Institutes of Biotechnology Malaysia, Jl. Bangi, Selangor, 43000, Malaysia; Department of Bioinformatics, School of Life Sciences, Indonesia International Institute for Life Sciences, Jl. Pulomas Barat Kav. 88, Jakarta, 13210, Indonesia; Department of Biology, Pace University, One Pace Plaza, 3rd Floor, New York, 10038, NY, United States of America

**Keywords:** ncRNA, gene regulation, Malpighiales, small RNA, RNAi

## Abstract

Rafflesiaceae is a family of endangered plants whose members are solely parasitic to the tropical grape vine *Tetrastigma* (Vitaceae). Currently, the genetics of their crosstalk with the host remains unexplored. In this study, we use homology-based *in silico* approaches to characterize micro-RNAs (miRNAs) expressed by *Sapria himalayana* and *Rafflesia cantleyi* from published omics data. Derived from secondary structures or hairpins, miRNAs are small regulators of gene expression. We found that some plant-conserved miRNA still exists in Rafflesiaceae. Out of 9 highly conserved miRNA families in plants, 7 families (156/157, 159/319, 160, 165/166, 171, 172, 390) were identified with a total of 22 variants across Rafflesiaceae. Some miRNAs were missing endogenous targets and may have evolved to target host miRNA, though this requires experimental verification. Rafflesiaceae miRNA promoters are mostly inducible by ethylene that mediates stress response in the host but could be perceived by the parasites as a signal for growth. This study provides evidence that certain miRNAs with ancient origins in land plants still exist in Rafflesiaceae, though some may have been coopted by parasites to target host genes.

## 1 Introduction

Rafflesiaceae is a family of endangered holoparasitic flowering plants known to produce the largest flowers in the world, and the only known plants to date to have lost their chloroplast genomes [1,2]. All three members *Sapria*, *Rafflesia*, and *Rhizanthes*, are solely parasitic to certain species of the plant genus *Tetrastigma* (Vitaceae), growing inside host tissues as clusters of endophytic cells and laying hidden until the time of flower development [3,4,5]. Germination of the seed within the host has never been observed, and it is unknown what host metabolites induce this process [6,7,8]. Inside the host, the embryo proliferates and spreads as the host cambium divides [4], with each cluster conceivably forming a mass of cells that give rise to clonal buds that extrude out of the host [6]. Depending on the species, it can take several months for the buds to develop and reach anthesis, with some flowers reaching a meter in diameter (*R. arnoldii*). However, the rarity of these holoparasitic plants – unique only to the dwindling forests of Southeast Asia and endangered status [9], compounded by their cryptic life cycle, recalcitrance to propagation [10], and extreme host specificity [11] make them incredibly challenging to study.

Genetic interactions between Rafflesiaceae holoparasites and their hosts remain largely unexplored. Evidently, Rafflesia has incorporated host genes through horizontal transfer, with up to 2% nuclear transcripts [12] and 40% mitochondrial genes [13] co-opted from the host. However, the exchange of small RNAs, specifically micro-RNA (miRNA), and the roles these molecules play in this parasitic system have not been elucidated.

Discovered nearly two decades ago [14,15], miRNAs are crucial regulators of gene expression, operating through gene silencing or RNA interference/RNAi [16]. miRNAs are approximately 22 nucleotides long and regulate gene expression by pairing with target genes and disrupting their function through cleavage or inhibition. Like their target genes, they are also differentially expressed. In plants, miRNA coding genes are transcribed by RNA polymerase II (Pol II) into longer primary miRNA (pri-miRNA). Enzymes fold the primary miRNA into pre-miRNA with stem-loop or hairpin structures, which are then eventually processed into shorter (c. 22 nt) mature miRNA that can pair and interfere with gene expression of their target transcripts [17]. miRNAs are essential for plant development and stress responses [18].

Parasitic plants are constantly communicating with their hosts, and in these communications, miRNA is known to be involved, for example, in the reciprocal delivery of miRNAs between *Cuscuta* spp. (dodder) and their respective hosts [19]. There are indications that miRNAs accumulate in the haustoria when *Cuscuta campestris* parasitizes *Arabidopsis thaliana* and *Nicotiana benthamiana* [20]. These trans-species miRNAs cause mRNA cleavage, secondary siRNA (small-interfering RNA) production, and decreased mRNA accumulation in the hosts, suggesting their role as a virulence factor. On the other hand, *N. tabacum* is able to genetically silence Dodder’s STM gene involved in the parasite’s formation of haustoria [21]. Regardless of host species, interface-induced miRNAs in *C. campestris* are consistently induced and also occur in *C. campestris* haustoria formed without the presence of a host [22]. A recent study identified trans-kingdom RNA silencing mechanisms involved in melon resistance to broomrape, highlighting miRNAs targeting disease resistance genes and uncovering pathways critical for host defense [23]. Similar mechanisms of miRNA exchange are also expected between Rafflesiaceae members and their hosts. Elucidating the genetics of these small regulatory molecules could yield basic insights on how to attenuate host immune response, for instance, to facilitate Rafflesiaceae parasitism that could benefit *ex situ* propagation applications and conservation efforts.

In this study, we aimed to characterize miRNA (the “hairpin”) in various members of Rafflesiaceae using *in silico* approaches on publicly available data (the “haystack”). These computational techniques have been successfully implemented in miRNA mining in other plant species [24,25,26]. *In silico* data mining can be especially useful for analyzing data from rare plants, as obtaining permits and samples for these plants can be challenging and costly. Nonetheless, a robust pipeline is essential to ensure the accuracy of the results [27]. In this study, we found evidence that some plant-conserved miRNA still exists in Rafflesiaceae members, though some may have been coopted by the parasites to target host genes. We discuss these findings in the context of the Rafflesiaceae–host parasitic relationship.

## 2 Materials and methods

### 2.1 Sequence data acquisition

**Figure 1:**
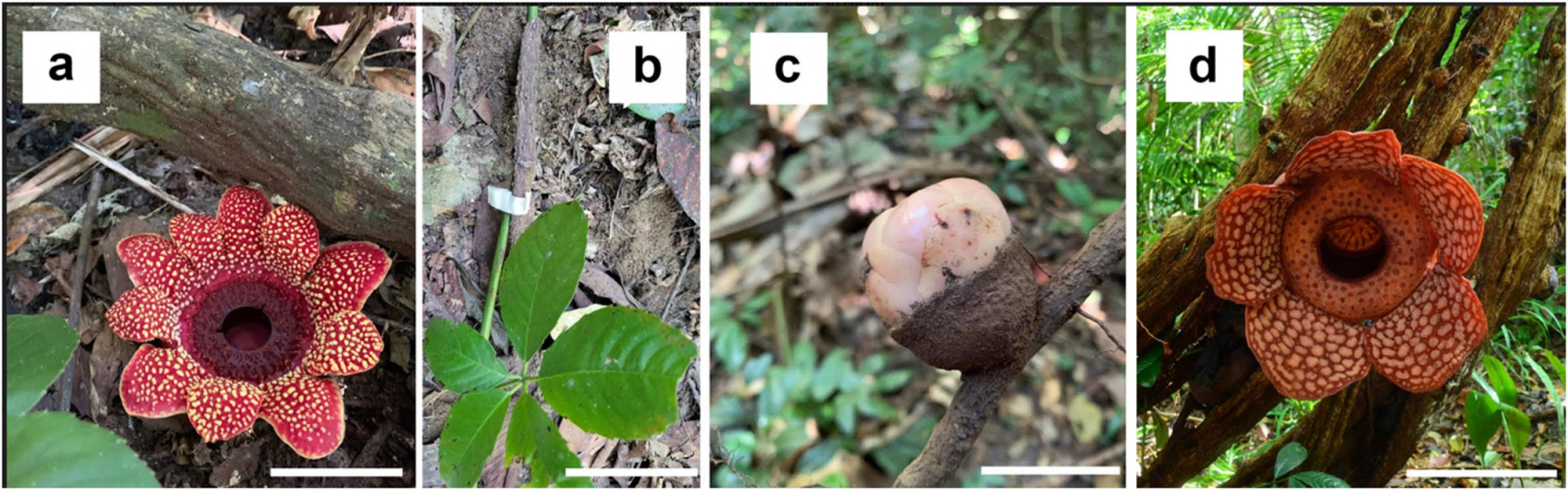
Rafflesiaceae plants in this study: *Sapria himalayana* (a) and its host, *Tetrastigma cauliflorum* (b), on which the *S. himalayana* bud (c) grows; *Rafflesia cantleyi* (d) attached to an unspecified host. Photo credit Adhityo Wicaksono (a and c), Jeanmaire Molina (b), and Siti Munirah Mat-Yunoh (d). Scale bars = 5 cm (a and b), 2 cm (c), and 30 cm (d).

We collected the following published omics datasets for analyses (plants see Figure 1): *Sapria himalayana* genomic sequences, transcriptomic/RNA-seq data from different tissues (bract, disc-stamen, inner perigone, outer perigone, and various sections of the flower bud, BioProject ID: PRJNA943542) [28], as well as RNA-seq data from *Rafflesia cantleyi* (BioProject ID: PRJNA378435 and PRJNA481608) including data from buds and flower [29]. The raw reads were trimmed with Trimmomatic v0.39 [30], and quality checked with FastQC v0.12.1 [31]. Once the adapter sequences and bad reads had been trimmed, the reads were ready for mapping or assembly.

We also submitted a sample of the uninfected root of *Tetrastigma cauliflorum* (a host species of *S. himalayana*) collected from Queen Sirikit Botanic Garden (QSBG, with permission from the National Research Council of Thailand) to Azenta Life Sciences (South Plainfield, NJ, USA) for standard RNA-seq service (using Illumina HiSeq 2x150bp). The coding sequences (CDS) *de novo* assembled from this RNA-seq data were used as the host plant miRNA target gene library for identified *S. himalayana* miRNAs. We also used the CDS of *Vitis vinifera* [32], the closest relative of *Tetrastigma* as a host proxy for target gene identification (described below). Moreover, the CDS for *Manihot esculenta* and *A. thaliana* [32] were also obtained for additional miRNA identification using BLAST, as described below.

### 2.2 Transcriptome mapping and *de novo* assembly

We conducted a *de novo* assembly of *S. himalayana* RNA-seq data and mapped these RNA-seq reads to its reference genome. Since no reference genome was available for *R. cantleyi* and *T. cauliflorum*, we performed *de novo* assembly on their RNA-seq data using Trinity v2.15.1 [33]. We used Galaxy Europe (https://usegalaxy.eu; The Galaxy Community 2022) pipelines of HISAT2 v2.2.1 [34] for mapping, StringTie v2.2.1 [35] or Salmon v1.10.1 [36] for transcript per million (TPM) value quantification, as well as bedtools v2.30.0 package [37] getFASTA to obtain the FASTA sequence of the mapped transcripts for miRNA identification. Transcriptome data were processed with TransDecoder v5.5.0 [38] to predict the CDS and peptide sequences within the transcripts. The predicted CDS and peptide sequences were then annotated with BLASTp and BLASTx via Diamond v2.0.15 [39] with the UniProtKB/SwissProt database [40,41] (update March 2023) and NCBI NR database (update July 28, 2023), with *e*-val cutoff at maximum 10^−5^. Further cross-checking was carried out with InterProScan v5.59-91.0 [42,43] with default databases (Pfam [44], PANTHER [45], SMART [46,47], and TIGRFAM [48]).

### 2.3 miRNA mining

To identify the miRNA, INFERNAL v1.1.4 [49] via Galaxy Europe (https://usegalaxy.eu) [50] was used. Rfam database v14.9 was used as the template for covariant models [38,51] and all miRNAs were sorted from all noncoding RNAs (ncRNAs) within the database. To identify possible convergently evolved miRNA between Rafflesiaceae and *Cuscuta* and *Orobanche*, stem-loop sequences of miRNA of *C. campestris* (30 miRNA) and *Orobanche aegyptica* (12 miRNA) from a previous study [20] were also converted into CM with CMBuild feature from INFERNAL package. CMSearch feature from the INFERNAL package was used to identify shim and rcan miRNAs from the genomic and transcriptomics sequences. The CMSearch was run twice for putative miRNA, applying either *e*-val < 1 × 10^−5^ to filter results, or using the “trusted cutoff” threshold in the model (http://eddylab.org/infernal/). Later, both results were compared, and the matching sequences were evaluated for variations and named using the miRNA nomenclature (*sensu* Zangishei et al. [20]) for each species. This resulted in precursor miRNA (with stem-loop/hairpin structures), from which, the mature miRNA sequences were identified.

To find additional miRNA in *S. himalayana*, we also blasted all miRNA hairpins (from https://mirbase.org/download/) against the assembled genome and transcriptomes of *S. himalayana* (max *e*-val < 1 × 10^−5^) using Geneious Prime (Biomatters, Ltd.) with results for “query centric alignment” to identify hairpins that have hits. These hits were then blasted against each of the CDS datasets: *A. thaliana*, *M. esculenta*, *V. vinifera*, *T. cauliflorum*, *S. himalayana*, and *R. cantleyi* and binned into “has hits” vs “no hits.” Those with “no hits” were collected and assumed to be non-coding RNAs that were then manually searched in PmiREN (Plant miRNA Encyclopedia [28]) to determine if the miRNA was conserved (i.e., with a significant hit of max *e*-val < 0.0001 against known plant miRNA). This workflow of finding additional miRNA was repeated for *R. cantleyi.*

To confirm the valid stem-loop miRNA sequences, sequences with no mature sequence identified were omitted. We also confirmed if the putative miRNAs were plant-based according to Rfam (https://rfam.org), RNAcentral (https://rnacentral.org), and miRbase (https://www.mirbase.org). Additionally, for *S. himalayana*, miRNAs predicted from the transcriptomic data were also cross-checked against its reference genome. We were unable to perform this for *R. cantleyi* which has no reference genome available yet. After all the putative miRNA were detected, alignment and hairpin secondary structures were predicted using RNAstructure at Dynalign Web Server (https://rna.urmc.rochester.edu/RNAstructureWeb/Servers/dynalign/dynalign.html) [52] at default settings. The images of the secondary structures were then merged, labeled, and had the mature sequences highlighted using Photoshop CS6 (Adobe). The TPM counts (transcript per million counts) of the miRNA genes were visualized using heatmaps generated by Heatmapper (http://www.heatmapper.ca/expression) [53].

### 2.4 miRNA promoter analysis

To analyze the promoter region for *cis*-acting regulatory elements of the genomic-identified miRNA genes, we extracted the 2k-bp upstream sequences of each miRNA gene and processed them using PlantCARE [54]. The target of PlantCARE elements comprises of three subjects: (1) phytohormones (ABRE, CGCTCA-motif, ERE, GARE-motif, P-box, TATC-box, TCA-element, TGA-element, and TGACG-motif), (2) abiotic and biotic stress responses (ARE, AT-rich sequence Box 4, G-box, GA, GATA, LTR, MBS, TC-rich repeats, and WUN motif), and (3) growth and development (CAT-box, circadian, GCN4-motif, MSA-like, MYB, and O2-site) [55].

### 2.5 miRNA target prediction

To identify the target genes for the resulting miRNA sequences, TargetFinder v1.7 [56] was used to predict miRNA targets based on complementarity scoring, using a threshold score of ≤4 to indicate high-confidence miRNA-mRNA interactions. CDS datasets for each species were utilized, and for *S. himalayana* and *R. cantleyi*, searches were performed against endogenous CDS as well as the host and proxy species CDS for cross-species targets. *S. himalayana* miRNAs (shim-miRNAs) were then searched against the CDS of *S. himalayana* (hereafter, “shim”) to identify endogenous genic targets, as well as searched against the CDS of *T. cauliflorum* (“tcau”) and of *V. vinifera* (“vvin,” host proxy) to determine genic targets of shim-miRNA in the host. Similarly, the same procedure was applied to other *R. cantleyi* (hereafter, “rcan”) miRNA against their respective CDS data to determine endogenous targets, as well as against tcau and vvin CDS to determine putative host targets.

## 3 Results

### 3.1 Rafflesiaceae miRNA

Out of 9 deeply conserved miRNA families in plants (Table 1; [27]), we characterized 7 miRNA families with a total of 22 variants in both *S. himalayana* and *R. cantleyi.* The miRNA family with the highest number of members is the mir159/319 family, with 3 members in *S. himalayana* and 4 members in *R. cantleyi* (see Tables 2 and 3 for details).

**Table 1:** Plant-conserved miRNA including Rafflesiaceae miRNA from this study

Family	*A. thaliana* (Dicot) (miRBase v22.1)	*Populus trichocarpa* (Dicot) (miRBase v9.2 *cit*. Axtel et al. [57])	*Oryza sativa* (Monocot) (miRBase v22.1)	*Selaginella moellendorffi* (Lycopod) (Axtel et al. [57] + miRBase v22.1)	*Physcomitrella patens* (Moss) (miRBase v9.2 + Axtel et al. [57])	*S. himalayana* (Dicot) (this study)	*Rafflesia cantleyi* (Dicot) (this study)
mir156/157	19	11	12	5	3	2	1
mir172	5		4			1	1
mir170/171	4	11	9	4	2	1	1*
mir165/166	9	17	14	3	13	2	2
mir159/319	6	15	8	2	5	4	4
mir396	2	7	9	1			
mir168	2		2				
mir160	3	8	6	2	9	1	
mir390	2	4	1		3	1	1

*Note: Truncated mir171_1 in *R. cantleyi* is excluded.

**Table 2:** List of identified and validated miRNAs in *S. himalayana* from the reference genome

miRNA Family	Rfam ID	miRNA	*S. himalayana* Stem-Loop miRNA Sequence	Length (nt)	*S. himalayana* Mature miRNA Sequence	Length (nt)
mir156	RF00073	mir156	**UGACAGAAGAGAGAGAGCUC**AACCCGGCAUUAACCUAAGAGAGUCUUGGUUAUGGUGGGAGUGUGCUCUUUCUUCUUCUGUCA	83	UGACAGAAGAGAGAGAGCUC	20
mir157	n/a	mir157	UG**UUGACAGAAGAUAGAGAGCAC**CGAUGAUGGCGUGCAAUAGUUGCAAACCAAUCAUUCGUGCUCTCUAGCTCCUGUCAUCAU	83	UUGACAGAAGAUAGAGAGCAC	21
mir159	RF00638	mir159a	GGCAGUUAGGUAGGGCUCCUUGACGUCCAAAUGAGGGUCUAAAUGAGCAGGGUAGCUGCCUAGUUAUGUgCUCCACGCUUCCACCCCGUCGAUGUAGUAAUAUGGGGGUAGGAUUGAGGAUUGCUUAGCCAGGGAGCUUUCCAACUCAUCUUUAAGUCUCUU**UUUGGAUUGAAGGGAGCUCUG**CUUCCUCUUUUC	195	UUUGGAUUGAAGGGAGCUCUG	21
		mir159b	GGCAGUUGGGUAGAGCUCCUUCAAGUCCAACAUAGGGUCUAACUGAGUAAGCAGGCUGCUUGGUUAUGGACUCCACAGUCCCGCUCCAUUGAAGcAGUGCUACCAGAGUAGGCUUGAGGAUUGCUUAGCCGGGGAGCUUUCUAACUCAAuUGUUAGCUCCUUU**UUUGGAUUGAAGGGAGCUCUA**CUUAACUCGUUU	196	UUUGGAUUGAAGGGAGCUCUA	21
		mir159c	UGCGGUGGGGUAGAGCUCCUCGAAGUCCAACAGAGGGUCUAACUGAGUCAGGUAGCUGCUUGGUUAUGGACUCCACCGUCCCACUCCAUCGAAUcUGCAUCAUGGGAGUAGGCUUGGGGGCCGCUUAGCCAGGGAGCUUCCAGCUCAACGUUAUAUCCAUU**CUUGGAUUGAAGGGAGCUCUA**CUUCCCCUUCUC	194	CUUGGAUUGAAGGGAGCUCUA	21
mir160	RF00247	mir160	CUG**UGCCUGGCUCCCUGUAUGCCA**UAUGCGGAGCCCAUUGAGAUGUCAAUAGUCUUCGUGGAUGGCAUAUGAGGGGCCAUGCAUAA	86	UGCCUGGCUCCCUGUAUGCCA	21
mir166	RF00075	mir166a	GUUGGUAGGAAUGUUGUUUGGCUCGAGGUCAUUUAGGUUCGacgccgcgauguggcgugccaggccgccuuuaucgucuuccaaaagaAAUUUAGGAUCAGUUCUCGUUAGGAAUCAUAAGUGAUC**UCGGACCAGGCUUCAUUCC**UGCCAAC	152	UCGGACCAGGCUUCAUUCC	19
		mir166b	UUUGAGAGGAAUGUUGUCUGGCUCGAAAACUUAGUUUCUUCAUGAUCCAGAUCAUCGUgcaccuguAGAUCUCACAGAUUUAUGGGUUCUUUUAGAUcUGUGUUG**UCGGACCAGGCUUCAUUCCCC**CCAAU	131	UCGGACCAGGCUUCAUUCCCC	21
mir171_1	RF00643	mir171_1	GUGUCACUUUGAUGUUGGCCCGGUUCACUCAGAGCGAGGCUAGGUUCUguuuuuuuuccuauuuuuauugguuacgaucauccuauGCCU**UUGAUUGAGCCGCGCCAAUAUC**UUAGUGAACCUU	124	UUGAGCCGCGCCAAUAUC	18
mir172	RF00452	mir172	CUGUUUGCUGGUGCAGCAUCUUCGAGAUUCACAAGCCUuuauuaggguuacagucACUGGGUUUCAGUCUUAAUUUAAUUUUAACACAGAAACCCUUUUUGUAUG**AGAAUCUUGAUGAUGCUGCAG**CGGCAAUGGG	136	AGAAUCUUGAUGAUGCUGCAG	21
mir319	RF03483	mir319	AAGGAGCUUUCUUCAGUUCAGUUCAUGGCAAGAAACAGCCUCAAAACUGCUGCUGAAUCGUUGGGUCAGGAACCCAUCAUCATCGUUUUUGAAUAAGGAAGGCUAGGUCGCGGCAAGCGAGAUGAGUUUAUGATCCAUCGAAGCAGGAGCUGUGUUAGGCUAUGCUUGUCGCGGC**UUGGACUGAAGGGAGCUCCC**U	196	UUGGACUGAAGGGAGCUCCC	20
mir390	RF00689	mir390	AGCAUGGAACAAUCCGUC**GAGCUCAGGAGGGAUAGCGCC**AUGAAUAAAAAUCGUGCUCGUCAGUUUUGUUCCGACGCUAUCUAUUCUGAGCUUGACAGCUUCUUCUUGCU	110	GAGCUCAGGAGGGAUAGCGCC	21
mir395	RF00451	mir395	AUGUCCCCUAGAGUUCCCUUGACCACUUCAUCGGGGACCUUCUUUAAUGGCUUCCUA**CUGAAGUGUUUGGGGGAACUC**CUGGUUCCAU	88	CUGAAGUGUUUGGGGGAACUC	21

Note: Bold-faced sequences in the stem-loop sequences refer to miRNA mature sequences.

**Table 3:** Validated miRNA families in *Rafflesia cantleyi* from the RNAseq data

miRNA family	Rfam ID	miRNA	*Ra. cantleyi* stem-loop miRNA sequence	Length (nt)	*Ra. cantleyi Mature* miRNA sequence	Length (nt)
mir156	RF00073	mir156	**UGUUGACAGAAGAUAGUGAGCA**CAGAUGAUGGCGUGCAAUGGAUGCAAACUAAUCAUUCGUGCUUUCUAGCUUCUGUCAUCA	82	UGUUGACAGAAGAUAGUGAGCA	22
mir159	RF00638	mir159a	UGCGAUUGGGUAGAGCUCCUUGACGUCCAACAAAGGGUCUAACUGAGUCAGGUAGCUGCUUGGUUAUGGAUUCCACCAUCCCACUCCAUUGAAUcUGUAUUAUGGGAGUAGGUUUGAGGAUUGCUUAGCCAGGGAGCUUUCUAACUCAUGGUUAUAUCCCUU**CUUGGAUUGAAGGGAGCUCUA**CUUCCGCUUCUC	195	CUUGGAUUGAAGGGAGCUCUA	21
		mir159b	GAUAGGGUAGAGCUCCUUGAAGUCCAACGUAGGGUAUAACUGAGUAAGAUAGUUGCUUGGUUAUGGACUCCACAGUCCAAUUCCAUCAAGAUGUGUAAUGGGAAUACGCUUGAGGAUUGCAUAGCGAGGGAACUUUCUUGCUCGUaGUUAUUUCUCUUC**UUUGGAUUGAAGGGAGCUCUA**CUUAUUUUCGUU	192	UUUGGAUUGAAGGGAGCUCUA	21
		mir159c	CGAGGCUGGGUAGAGCUCCUUGAAGUCCAACAUAGGAUCUGACGGAgCAAGCGAGCUCCUUGGUUAUGGACUCCACAGUCCCACUCCACCGAAGCUGCGCAUGGGAGUUGGCUUGAGGAUUGCUUAGCCAUGGAGCUUUCUAACUCGUCGUUAAAUCCCGU**UUUGGAUUGAAGGGAGCUCUA**CUUCCUCUUUCU	194	UUUGGAUUGAAGGGAGCUCUA	21
		mir159d	GGCGAUAGGGUAGAGCUCCUUGAAGUCCAACGUAGGGUAUAACUGAGUAAGAUAGUUGCUUGGUUAUGGACUCCACAGUCCAAUUCCAUCAAGAUGUGUAAUGGGAAUACGCUUGAGGAUUGCAUAGCGAGGGAACUUUCUUGCUCGUaGUUAUUUCUCUUC**UUUGGAUUGAAGGGAGCUCUA**CUUAUUUUCGUU	195	UUUGGAUUGAAGGGAGCUCUA	21
mir166	RF00075	mir166a	UUUGAGAGGAAUGUUGUCUGGCUCGAAAACUUAAUUUCUUCAUGAUCCAGAUCAUCGCCUUCcuguAGAUCUCACAGAUUUAUGGGUUCUGUUAGAUcUGUGUUG**UCGGACCAGGCUUCAUUCCCC**CCAAU	131	UCGGACCAGGCUUCAUUCCCC	21
		mir166b	UUUGAGGGGAAUGUUGUUUGGUUCAAGCAACCCGUUCGAUCGGAUCGAGUGGGUUCCCAUUUGGCUACAUUUC**UCGGACCAGGCUUCAUUCCCC**ACGAA	99	UCGGACCAGGCUUCAUUCCCC	21
mir171_1	RF00643	mir171_1	UAAUAAGUAAGGUAUUGGCGCGCCUCAAUCCACUUGCUUUGGUCUUCGauuguuCGCCUGGUUGAAAGUAAGUUA**GAUUGAGCCGCGCCAAUAUC**UGACUUUUACUG	107	GAUUGAGCCGCGCCAAUAUC	20
		mir171_1t*	GUGUCACUUUGAUGUUGGCCCGGUUCACUCAGAGCGAGGCUAGGUUCUguuuuuuucauauuuuuauugguaacgaucauccuacGCCUUU**GAUUGAGCCGCGCCAAU**	108	GAUUGAGCCGCGCCAAU	17
mir172	RF00452	mir172	CUGUUUGCUGGUGCAGCAUCUUCGAGAUUCACAUACCUuuauuaacguuacaguUAUGGGAUUCAGUCUUAAUUUCAUUUUGACACAGAAACCGUUUUUGUAUG**AGAAUCUUGAUGAUGCUGCA**GCGGCCAUGAG	135	AGAAUCUUGAUGAUGCUGCA	20
mir319	RF03483	mir319	GGAGCUUUCUUCAGUUCAGUUCAAGGCAGAAACAGCUUAAAAACUGCUGCUGAAUCGUUGGGUCACGAACACAUCAUCUUUUGAAGAAGAGAUACUUGGUAGCGAGAAGCGAGAUGUGUUUUUGAUCCAUCGAAGCAGGAGCUGAGUUGGGCUAUGCUUGUCGCGG**CUUGGACUGAAGGGAGCUCC**	186	CUUGGACUGAAGGGAGCUCC	20
mir390	RF00689	mir390	UGGAGUAAUCUGUUG**AGCUCAGGAGGGAUAGCGCC**AUGAAUAAAAAUCUUGCUCGUGAGuuuuguuccGACGCUAUCUAUUCUGAGCUUUACGGCUUCUUCUU	103	AGCUCAGGAGGGAUAGCGCC	20

Note: Sequence marked with “t” and asterisk (*) is truncated and excluded from the structural prediction.

**Figure 2:**
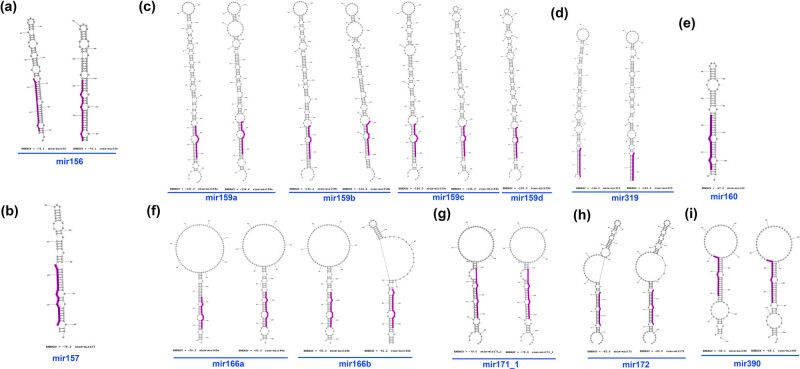
The identified miRNA precursor stem-loop minimal free energy structures with the mature miRNA sequences marked in purple in both shim and rcan. shim-mir395 is not shown as it is based on genomic evidence. miRNA: mir156 (a), mir157 (b), mir159 (c), mir319 (d), mir160 (e), mir166 (f), mir171_1 (g), mir172 (h), and mir390 (i).

Among the miRNA hairpin structures in Rafflesiaceae (Figure 2), the mir159/319 family has relatively longer stem structures (Figure 2c), while mir166, mir171_1, mir172, and mir390 families have notably large loops (Figure 2f–i).

**Figure 3:**
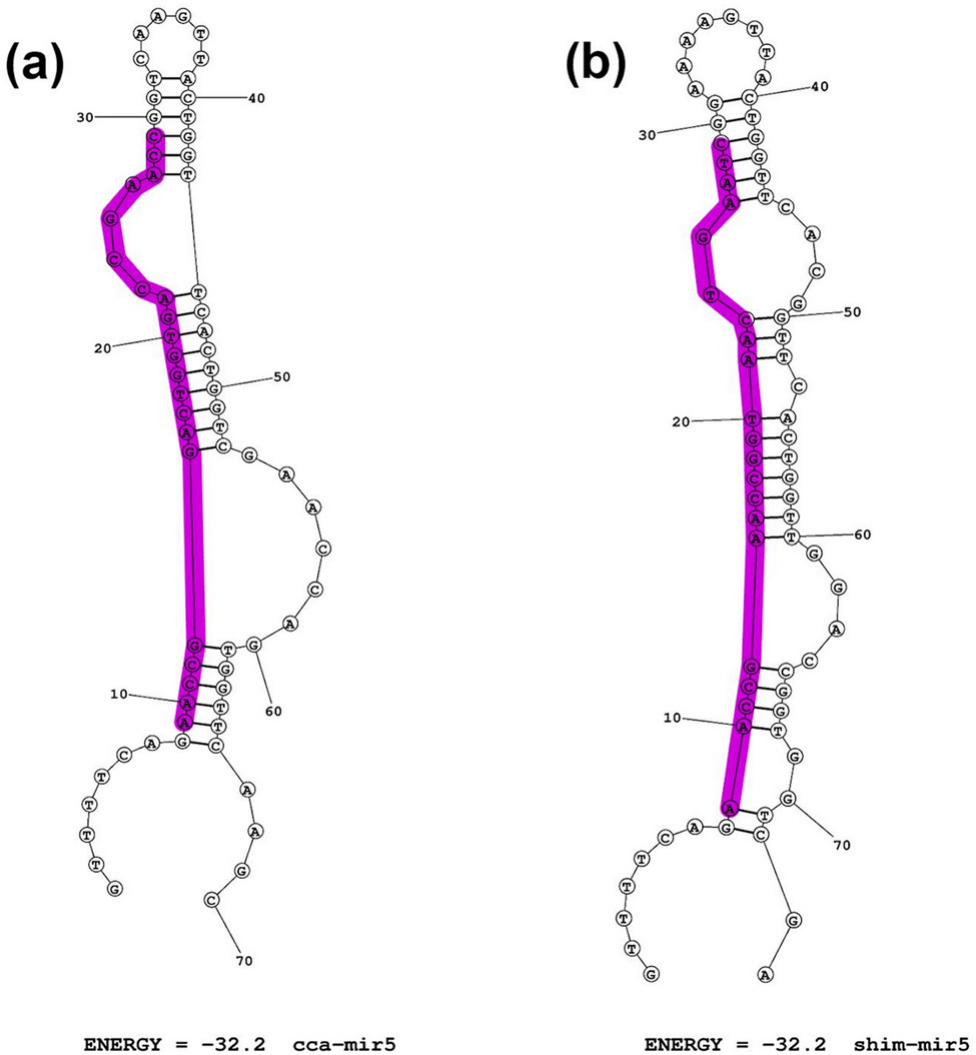
The identified miRNA precursor stem-loop structures for shimmir5 next to cca-mir5 from Zangishei et al. [20] with the mature miRNA sequences marked in purple. mir5: *C. campestris* (cca) (a) and *S. himalayana* (shim) (b).

A putative convergently evolved miRNA, shim-mir5, similar to cca-mir5 [20] was also detected in *S. himalayana* (Figure 3). However, we did not find potential endogenous nor host targets for shim-mir5.

### 3.2 Rafflesiaceae miRNA targets

miRNAs bind to specific gene targets to regulate gene expression. Using TargetFinder against respective CDS, we predicted potential endogenous (shim, rcan) and host targets (tcau, vvin) (Table 4). The transcriptome data for tcau (total of 181,320,714 reads totaling 54,396 Mb, with 90.04% bases with *Q* score ≥30) were *de novo* assembled as described above. CDS sequences were then retrieved from this and used for host target prediction in Targetfinder, though CDS for vvin, which is better annotated, was also used if there were no targets found using tcau CDS. mir171_1 was predicted to consistently target *Scarecrow-like protein* (*SCL*) in all species. mir159/mir319 and mir390 have the same endogenous targets: MYB and YfaU, respectively in both shim and rcan. However, mir390 has a different target in the host proxy (*LRR RLK, MIK2*). There were also instances when either shim or rcan has the same target as the host (or host proxy). For example, mir156, mir166, and mir172 present the same target for rcan and vvin.

**Table 4:** Putative target genes of shim and rcan miRNA (endogenous and in host tcau and in host proxy vvin). Only the highest-scoring targets are indicated. *mir395 was predicted from genomic evidence. n/a = no target found

miRNA	Targets in			
	*S. himalayana* (shim)	*R. cantleyi* (rcan)	*T. cauliflorum* (tcau)	*V. vinifera* (vvin)
mir156	*CSL D1*	*SPL*	n/a	*SPL*
mir157	n/a	n/a	n/a	*SPL*
mir159	Ty3-G, *MYB*, non-LTR retrotransposon reverse transcriptase	*MYB*, Networked 1D	TNT, *SRP72*	Ty3-G, *MYB*, SPOROCYTELESS, PUMILIO 24
mir160	n/a	n/a	n/a	*ARF*
mir166	n/a	*HD-Zip*	n/a	HD-Zip
mir171_1	*SCL*	*SCL*	*SCL*	*SCL*
mir172	n/a	*RAP2-7/AP2*	n/a	*RAP2-7/AP2*
mir319	*MYB*	*MYB*	n/a	*UNE12*
mir390	YfaU	YfaU	n/a	*LRR RLK, MIK2*
mir395*	n/a	n/a	n/a	APS1

### 3.3 miRNA expression levels

**Figure 4:**
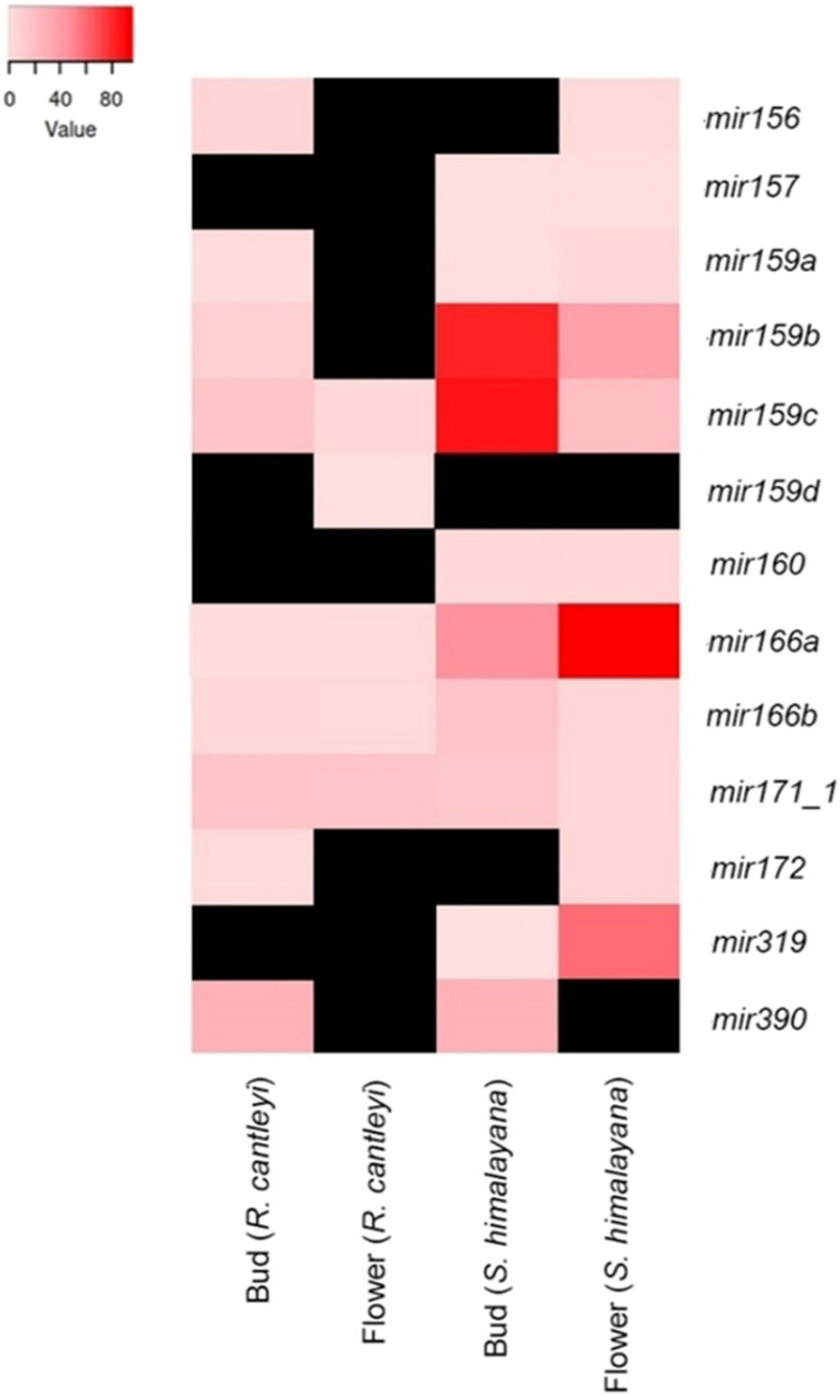
The TPM values for each miRNA for both *S. himalayana* and *R. cantleyi*. The gradient of white (zero) to red (high) shows the degree of expression (black not found/not applicable).

miRNA showed differential expression between bud and flower stages (Figure 4). Though expression was missing for certain miRNA (black), mir159 was slightly more upregulated in buds for both rcan and shim. mir166 was detected in both buds and flowers of both rcan and shim, with shim-mir166a having a more pronounced expression in the flower. mir171_1, though expressed in both species, had relatively low expression in both stages.

### 3.4 Features of miRNA gene promoters

**Figure 5:**
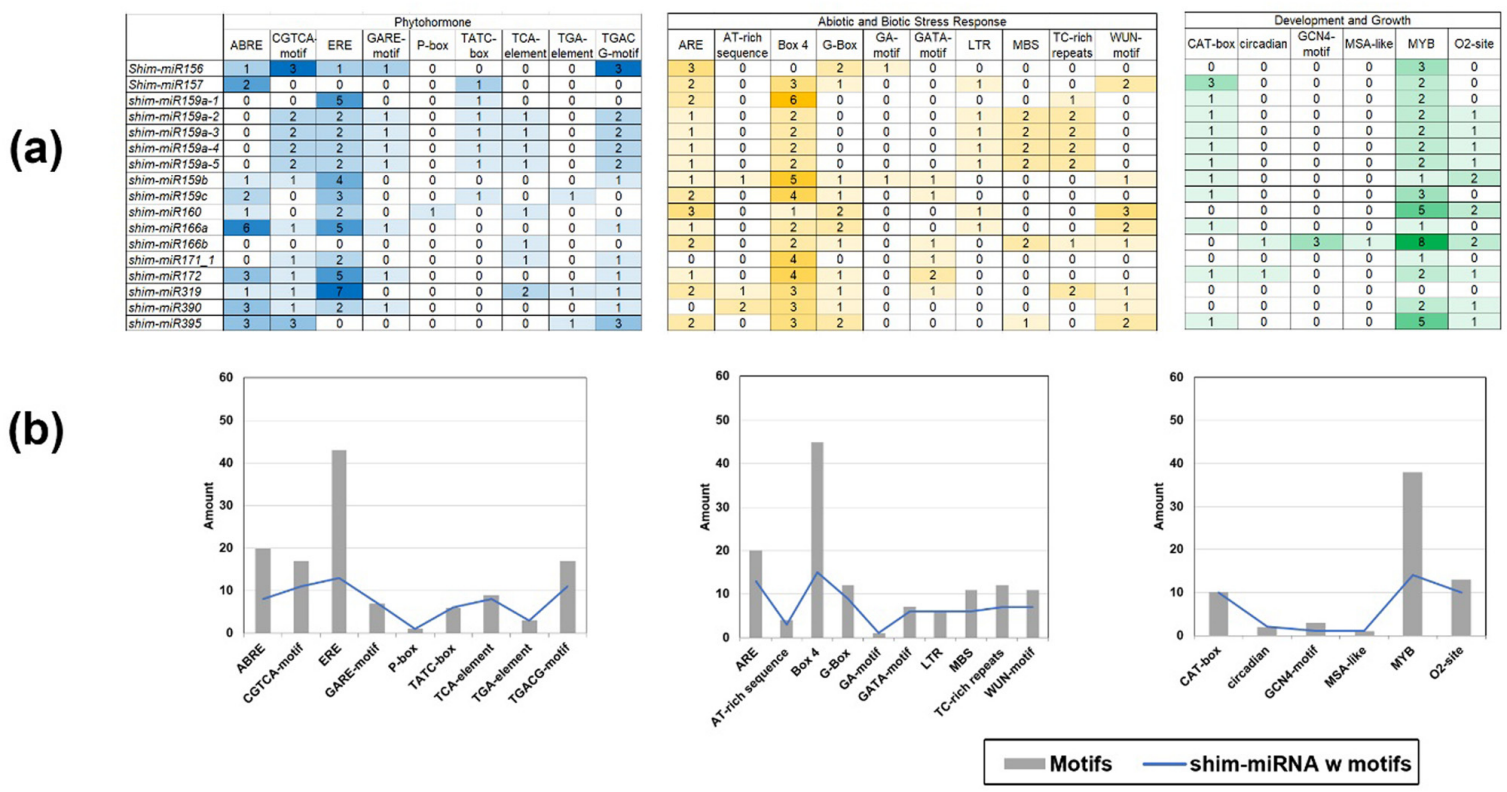
The identified features of the *S. himalayana* miRNA gene promoters located 2k bp proximal to the gene (a). The detected *cis*-acting regulatory elements or CARE motifs were summed (b, gray bars) to show which motifs are highly represented on each miRNA gene. The number of the detected miRNA with the motifs was also summed (b, blue lines).

The *cis*-acting elements for phytohormonal influence, stress and environmental responses, and developmental responses (Figure 5) were analyzed for shim, which has its reference genome published [28]. It appears that the shim-miRNA gene promoters were dominated by ethylene response elements (ERE), light-responsive Box-4 elements, and MYB transcription factor-related elements.

## 4 Discussion

### 4.1 Plant-conserved miRNAs exist in Rafflesiaceae

miRNA has been considered molecular taskmasters, regulating many biological processes through gene silencing or RNA interference/RNAi [16]. Out of 9 highly conserved miRNA families in plants [27], we identified 7 families (156/157, 159/319, 160, 165/166, 171, 172, 390) with 22 variants (total 12 miRNAs found in *S. himalayana* and 10 in *R. cantleyi*; Tables 1–3; Figure 2). In addition, we recovered miR395 (from shim). This number of miRNA families is comparatively small compared to photosynthetic plants. Between *Arabidopsis* and *Oryza*, 91 potentially conserved miRNAs have been identified [58]. Our homology-driven methods identified conserved miRNAs but likely missed novel ones specific to Rafflesiaceae. Between the holoparasites *C. campestris* and *Orobanche aegyptiaca*, the same conserved miRNAs detected in Rafflesiaceae were also found, though there were a few more, such as miR164, miR168, miR396, and miR398, that were present in both *C. campestris* and *O. aegyptiaca* (and other photosynthetic plants [20]) but lacking in Rafflesiaceae. It is possible that our *in silico* methods may not have mined all plant-conserved miRNAs in Rafflesiaceae, but the evolutionary loss of these miRNAs due to their unique life cycle may be an alternate explanation. Cai et al. [2] reported that 44% of genes conserved in eurosids were lost in *Sapria*, as a result of genome streamlining or the tendency toward reduction in non-coding DNA which has been documented in many obligate parasites, whether bacterial or eukaryotic.

Each miRNA family has a different hairpin structure. For instance, the miR159/319 family in this study has the longest stem structures, while miR166, for example, has a shorter stem with much bigger loop structures. Unfortunately, the reason behind the diversity in hairpin size is still not yet explained. Hypothetically, a longer hairpin might prolong its existence in the cytoplasm before being cleaved into mature miRNA, as its structure would be thermodynamically more stable [59] or a long hairpin RNA by itself could act as RNA silencing agent [60]. The shorter hairpin, on the other hand, would be immediately processed to produce a mature miRNA sequence. This would require more tests, including 3D modeling followed by miRNA–mRNA docking simulation, and molecular dynamics to confirm the structural stability [61,62].

### 4.2 Putative genic targets of detected miRNA

Though conserved miRNAs were characterized, not all Rafflesiaceae miRNAs were found to have endogenous targets and may have evolved to target host miRNA, though this requires experimental verification. Parasites have been shown to synthesize and deliver miRNAs that target mRNA in their host primarily to subvert the host immune response [63]. For some miRNA, we found internal targets (i.e., within Rafflesiaceae) that were annotated similarly to host targets (Table 4), and we think that in these cases, the miRNA is involved in endogenous genetic regulation of the parasite itself, rather than the host. For example, miR156/157 and miR165/166 were recovered from non-infective portions of *C. campestris* [20], suggesting these are probably involved in endogenous genetic regulation of the parasite. The same two miRNA families were recovered in Rafflesiaceae. In addition, we characterized miR171 and miR172 in Rafflesiaceae, whose targets were similar in both host and parasite.

In rcan, miR156/157 targets *SQUAMOSA PROMOTER BINDING PROTEIN-LIKE (SPL)* family involved in leaf/root development, flowering, and stress response [64]. Since we did not detect a genic target in tcau, perhaps due to poor gene annotation, we explored vvin as host proxy and identified the same genic target: *SPL*. miR156 delays flowering by targeting SPL transcription factors, while miR172 has the opposite effect, promoting flowering by depleting Apetala2/AP2 [64,65]. Both of these miRNAs conceivably interact with one another to regulate flowering in Rafflesiaceae. However, the internal target of miR156 in shim was *CSL D1* (cellulose synthase-like D1 protein) which regulates plant organ size through cell division and has been found to be highly expressed in immature tissues [66], which may explain the limited expression in the mature shim flower (Figure 4), though absent in its bud. The absence of expression of certain miRNA (Figure 4, black) could be an artifact of limitations in data quality and/or computational miRNA mining approaches.

Though we did not find an internal target for miR172 in shim, in rcan, miR172 potentially targets *RAP2-7*, a member of *Apetala2/AP2* involved in flowering regulation and innate immunity (The Arabidopsis Information Resource/TAIR). In vvin (none found in tcau), *RAP2-7* was also detected as a target. RAP2-7 is an ethylene-responsive transcription factor, which confers a delay in flowering time and is upregulated during pathogen attack [41]. miR172 expression in rcan and shim (Figure 4), and consequent RNAi of RAP2-7 may be a mechanism to control the parasite’s flowering, while trans-species regulation could suppress host immunity.

miR166 also had similar genic targets in rcan and in vvin (none found in shim and tcau) – homeobox-leucine zipper protein (HD-zip) *ATHB-15* which regulates vascular development in the inflorescence [67]. Thus, higher expression of this miR166 in both shim and rcan flower (vs bud, Figure 4) may be indicative of increased regulation of xylem development in this stage [68]. The putative genic target of miR171 for all taxa examined here (shim, rcan, tcau, vvin) was *SCL* (scarecrow-like). Overexpression of miR171 and concomitant silencing of *SCL* genes resulted in *Arabidopsis* and rice showing branching defects and late flowering suggesting conserved function of miR171 in plants [69]. This miRNA also regulates various plant responses including phase transitions, somatic embryogenesis, hormone signaling, and stress responses [70] which may explain the expression of miR171 in both bud and flower, though more so in the flower of both species, shim and rcan (Figure 4).

miR159 and miR319, which are related in origin but considerably diverged in function [71] were also detected in shim, but only miR159 was identified in rcan. Genic targets include members of *MYB* (miR159) and *UNE12*, a type of TCP/TEOSINTE BRANCHED1/CYCLOIDEA/PROLIFERATING CELL FACTOR (miR319), which are involved in male function and leaf development/hormone synthesis [64,72] respectively. Greater expression of miR159 in shim and rcan buds (vs flowers, Figure 4) suggests repression of *GAMYB* (gibberellin-induced MYB) and possible attenuation of male development [73]. Though miR319 targets *MYB* in both rcan and shim, another potential genic target in shim is “unnamed protein product” with the top blast hit “non-LTR retrotransposon reverse transcriptase from *Cuscuta epithymum*.” Since this was not recovered as a target in rcan, it is not clear if this is a case of convergent evolution in holoparasites. >70% of genic targets in *Cuscuta* are involved in silencing transposable element (TE) expression [20]; thus, it is not unlikely that shim-miR159 has evolved this new TE-related target. A transposon protein was also retrieved as a target in tcau for miR159 (Retrovirus-related Pol polyprotein from transposon TNT), as well as in vvin (Ty3-G Gag-Pol) in addition to MYB. In rcan, another putative internal target was Networked 1d/kinase interacting (KIP1-like) which is a pollen protein [74], and thus relevant to the expected target of miR159 with respect to male development.

miR160 was detected in shim but not in rcan. This miRNA targets auxin response factors (*ARF*), which are involved in multiple stages of plant development including embryo, leaf, root, flower, and seed development. No endogenous target was found in shim and in tcau. The lack of a target in shim could indicate differential regulation in host and parasite, perhaps allowing shim to escape miR160 auxin regulation facilitating the development of the flower’s giant size. In the host proxy vvin, miR160 targets were *ARF17* and *ARF18*, involved in anther dehiscence [75] and repression of *AGAMOUS* that controls stamen-petal organ specification [76], respectively.

The plant conserved miR390 was also identified in both shim and rcan buds (Figure 4) targeting the specific enzyme 2-keto-3-deoxy-l-rhamnonate aldolase (YfaU) endogenously, but in the host proxy vvin (none found in tcau) genic targets include *MDIS1-interacting receptor-like kinase 2 (MIK2)* and probable *LRR receptor-like serine/threonine-protein kinase (LRR RLK).* These identified targets differ from the expected targets of TAS3 implicated in ARF repression and indirect miR165/166 regulation [64]. YfaU is an enzyme that specifically catalyzes the reversible retro-aldol cleavage of 2-keto-3-deoxy-l-rhamnonate to pyruvate and lactaldehyde [41] and is involved in the rhamnose catabolic pathway. Rhamnose sugars are commonly found in plant pectins. MIK2 and LRR RLK are involved in the activation of plant immune response against various pathogens [77]. The disparate targets of this miRNA imply different genetic regulatory mechanisms, one that prevents breakdown of rhamnose in the parasite, which may be important for infection as observed in plant pathogens [78,79] while concomitantly disrupting immune response in its host.

### 4.3 miRNA in plant parasites

Like *Cuscuta*, Rafflesiaceae seemed to have lost multiple conserved miRNAs of core eudicots (miR164, 167, 168, 169, 394, 396, 397 [80]) involved in leaf and root development and immune resistance [64]; these processes rendered obsolete by the parasitic lifestyle [20]. Though *Cuscuta* was missing miR395, we found genomic evidence for miR395 in shim, albeit missing in its transcriptome. Though there was no endogenous target found in shim, we detected ATP sulfurylase 1 (APS1, Table 4) as a target in the host proxy vvin. We speculate that by inhibiting host APS, which promotes sulfur uptake and assimilation [81], it allows the accumulation of metabolically essential sulfate in host shoots [82] to which *Sapria* is attached.

Novel miRNA may arise from *de novo* emergence or neofunctionalization or horizontal gene transfer [20]. We attempted to determine if miRNAs that Zangishei et al. [20] identified as novel in *C. campestris* are present in Rafflesiceae, as a result of convergent evolution. This was motivated by their finding that there were some new miRNAs identified in *Cuscuta*, for example, Ccamp-miR15 with a sequence similar to more related *Solanum lycopersicum*, and even in the more distant *Oryza sativa*. We found a homolog in shim (shim-mir5, Figure 3) to cca-mir5 characterized by Zangishei et al. [20] in *C. campestris*, but we did not find an endogenous target, nor potential host targets (results not shown).

The identified miRNA promoters in *S. himalayana* contained multiple ethylene-responsive-binding elements (ERE, Figure 5). Ethylene, a stress response mediator produced during biotic and abiotic stress (e.g., pathogens, drought, or heat) [83], may act as a growth signal for the parasite. This could explain the increased presence of ERE in Rafflesiaceae miRNA promoters, potentially reflecting an evolutionary adaptation to host-derived ethylene during parasitism. Interestingly, the finding that ethylene-reception mutants of the parasitic plant *Phtheirospermum japonicum* are unable to invade host roots [84] lends credence to this hypothesis in *Sapria*, which may have convergently evolved to recognize ethylene as a growth signal. In addition to ERE, light-responsive elements (BOX4, Figure 5) were identified in Rafflesiaceae miRNA promoters, implying phototropic response in Rafflesiaceae [85]. As expected, like any other plant, motifs for the large family of MYB were abundant in shim miRNA promoters (Figure 5) as these transcription factors are involved in various plant processes including biotic and abiotic stress responses, development, differentiation, and metabolism [86].

### 4.4 Limitations and future studies

This study provides a foundation for understanding miRNA roles in parasitism within Rafflesiaceae. However, given that miRNA covariance models were derived from the Rfam database and previous studies, further research will be essential to identify putative novel miRNAs specific to Rafflesiaceae species. This includes expanding research beyond *R. speciosa* and *S. himalayana* to other Rafflesiaceae members. Additionally, experimental validation through small RNA sequencing at the host-parasite interface will be crucial to confirm the miRNAs discovered here and to identify any novel miRNAs that might play unique roles in parasitism.

Despite these limitations, our findings reveal a subset of conserved plant miRNAs in *R. speciosa* and *S. himalayana*, highlighting similarities between these parasitic plants, as well as in comparison to their host plants. These insights serve as an important preliminary step toward understanding miRNA evolution and function in parasitic plants, setting the stage for deeper explorations into the molecular mechanisms underlying Rafflesiaceae-host interactions.

## 5 Conclusion

In this study, we used homology-based *in silico* approaches to characterize conserved miRNAs in Rafflesiaceae from published omics data. Though this approach limited us from characterizing novel miRNA that may have evolved because of its specialized parasitic relationship with Tetrastigma, our study provided confirmation that certain miRNA that have ancient origins in land plants [80] and are also present in Rafflesiaceae. Despite the unique adaptations of Rafflesiaceae to a parasitic lifestyle, they retain a subset of miRNAs commonly found in non-parasitic plants, such as mir156, mir159, and mir166, which likely contribute to essential regulatory functions. Differential expressions across developmental stages further indicate that miRNAs may help coordinate growth and interaction with host plants. Small RNA sequencing at the host–parasite junction could confirm the miRNA characterized in this study, as well as shed light on the cryptic genetics that underlie the development of the world’s largest flowers, including how these unique miRNAs are involved in gene silencing/RNAi of host genes to facilitate and sustain Rafflesiaceae infection. Future studies exploring novel miRNAs unique to these species may yield insights into the evolution and specialization of parasitic plants.
